# Identification of Key Pathways Associated With Residual Feed Intake of Beef Cattle Based on Whole Blood Transcriptome Data Analyzed Using Gene Set Enrichment Analysis

**DOI:** 10.3389/fvets.2022.848027

**Published:** 2022-04-18

**Authors:** Godstime A. Taiwo, Modoluwamu Idowu, James Denvir, Andres Pech Cervantes, Ibukun M. Ogunade

**Affiliations:** ^1^Division of Animal and Nutritional Science, West Virginia University, Morgantown, WV, United States; ^2^Department of Medicine, Surgery, and Biomedical Sciences, Joan C. Edwards School of Medicine, Marshall University, Huntington, WV, United States; ^3^Agricultural Research Station, Fort Valley State University, Fort Valley, GA, United States

**Keywords:** protein metabolism, cellular response, feed efficiency, oxidative stress, heat stress

## Abstract

We applied whole blood transcriptome analysis and gene set enrichment analysis to identify pathways associated with divergent selection for low or high RFI in beef cattle. A group of 56 crossbred beef steers (average BW = 261 ± 18.5 kg) were adapted to a high-forage total mixed ration in a confinement dry lot equipped with GrowSafe intake nodes for period of 49 d to determine their residual feed intake (RFI). After RFI determination, whole blood samples were collected from beef steers with the lowest RFI (most efficient; low-RFI; *n* = 8) and highest RFI (least efficient; high-RFI; *n* = 8). Prior to RNA extraction, whole blood samples collected were composited for each steer. Sequencing was performed on an Illumina NextSeq2000 equipped with a P3 flow. Gene set enrichment analysis (GSEA) was used to analyze differentially expressed gene sets and pathways between the two groups of steers. Results of GSEA revealed pathways associated with metabolism of proteins, cellular responses to external stimuli, stress, and heat stress were differentially inhibited (false discovery rate (FDR) < 0.05) in high-RFI compared to low-RFI beef cattle, while pathways associated with binding and uptake of ligands by scavenger receptors, scavenging of heme from plasma, and erythrocytes release/take up oxygen were differentially enriched (FDR < 0.05) in high-RFI, relative to low-RFI beef cattle. Taken together, our results revealed that beef steers divergently selected for low or high RFI revealed differential expressions of genes related to protein metabolism and stress responsiveness.

## Introduction

Residual feed intake (RFI), a measure of feed efficiency, continues to be of great economic importance due to increasing cost of animal feeds (1). Residual Feed Intake is the difference between an animal's actual feed intake and its predicted feed intake for a given level of maintenance and body weight gain (1). Feed efficient animals consume less than expected and have a low (negative) RFI, while inefficient animals consume more than expected and have a high (positive) RFI. Thus, beef cattle selected for low RFI have decreased feed costs because they consume less dry matter when compared with high-RFI beef cattle while maintaining similar growth performance.

Due to the great economic importance of RFI, the biological mechanisms underlying variation in this trait have always been of great interest; however, these mechanisms have not been fully understood. Difference in RFI has been suggested to be an indication of differences in metabolism rather than differences in growth performance because the trait is phenotypically independent of growth performance (1). Several studies have applied whole transcriptome analysis of several tissues such as liver and ruminal epithelium to further understand the biological mechanisms regulating feed efficiency traits including RFI in beef cattle (2–4). For instance, Kong et al. (3) analyzed the rumen epithelial transcriptome from low-RFI and high-RFI beef steers and observed increased tissue morphogenesis and greater expression of mitochondrial genes in low-RFI compared to high-RFI steers. Mukiibi et al. (4) analyzed liver tissue transcriptome profile and observed differential expressions of genes involved in nutrient metabolisms and cellular development in beef steers divergent for low and high RFI. However, these studies involve invasive sample collection procedures. Despite the convenience of collection and relatively non-invasive accessibility of blood in ruminants, very few attempts have been made to apply whole-blood transcriptome to understand the biological mechanisms associated with RFI in animals. Genes expressed in peripheral blood cells have been demonstrated to reflect physiological changes in different body tissues and can highlight biological processes related to overall metabolisms. Therefore, the objective of this study was to analyze the whole-blood transcriptome data of beef steers via gene-set enrichment analysis to identify key pathways associated with divergent selection for low or high RFI in beef cattle.

## Materials and Methods

### Animals and Sample Collection

A total of 56 crossbred growing beef steers with average BW of 261.3 ± 18.5 kg were fed a high-forage total mixed ration (TMR; primarily consisting of triticale silage; rye grass silage; and a ration balancing Supplementary Table 1) in five confinement dry lot pens (15 by 47 m^2^), each served by three GrowSafe 8000 (GrowSafe Systems Ltd., Airdrie, Alberta, Canada) feeding nodes to measure individual feed intake and two In-Pen Weighing Positions (IPW Positions, Vytelle LLC) to measure daily BW for a total of 49 d after 15-d adjustment period to the feeding facilities. The use of IPW Positions has enabled the measurement of individual animal BW multiple times in a day (5). The IPW Positions measure the partial BW by weighing the front end of an animal (6). The IPW positions were positioned at a water trough in each pen such that an animal must place its front feet on the scale in order to drink (7). The partial BW of the animals was measured every second the animals stayed on the scale while drinking. More details on the accuracy, use and applicability of IPW positions have been described in previous studies (5, 6). In this study, approximately 617 ± 92 daily BW data points (after filtering outliers) per animal were generated and were regressed on time to calculate beginning BW, mid-test BW, and average daily gain (ADG) of each animal. Animal ADG and metabolic mid-test BW (mid-test BW^0.75^) were regressed against individual average daily intake, and RFI was calculated as the residual or the difference between the predicted value of the regression and the actual measured value based on the following equation: Y = β_0_ + β_1_X_1_ + β_2_X_2_ + ε, where Y is the observed DMI (kg/d), β_0_ is the regression intercept, β_1_ and β_2_ are the partial regression coefficients, X_1_ is the mid-test metabolic BW (kg), X_2_ is the ADG (kg/d), and ε indicates the RFI [kg/d; (8)]. After the determination of RFI values for all animals, the most-efficient with the lowest RFI (low-RFI; *n* = 8) and the least-efficient with the highest RFI (high-RFI; *n* = 8) beef steers were selected, kept separate from others, and kept on the same diet for additional 21 d (designated in this study as d 50–70). On d 56, 63, and 70, 10 mL of blood samples were collected before morning feeding into tubes containing sodium heparin. Immediately after collection, subsamples (500 μL each) were transferred into RNA-protect tubes (Cat. No. 76554; Qiagen) containing a reagent that lyses blood cells and stabilizes intracellular RNA and stored at −80°C until later analysis.

### RNA Extraction, Library Preparation, and Sequencing

Prior to RNA extraction, whole blood samples collected on d 56, 63, and 70 were composited for each steer. Total RNA was extracted from the composited samples using RNeasy Protect Animal Blood kit (Cat. No. 73224; Qiagen) following the manufacturer's instructions. RNA concentration was measured using a NanoDrop One C spectrophotometer with an A260:A280 ratio from 1.8 to 2.0 (Thermo Fisher Scientific, Waltham, MA, USA). All RNA samples had RNA integrity numbers >8.0. Dual indexed RNA Libraries were prepared from 100 to 250 ng of total RNA per sample using the KAPA RNA HyperPrep Kit with RiboErase (Human, Mouse, Rat) Globin Reduction method in the WVU Genomics Core according to the kit manufacturer's instructions. Library quality was assessed by electrophoretic analysis on the Agilent 4,200 TapeStation system with High Sensitivity D1000 screentape. RNA libraries were sequenced in a dual indexed 2 × 50 paired-end run on an Illumina NextSeq2000 equipped with a P3 flow.

### Data and Statistical Analysis

For the RNA-seq data, reads were trimmed using Trimmomatic v 0.39 to remove low-quality base calls and adapter sequences (9), and then aligned to the Bovine reference genome ARS-UCD1.2 (10) using HISAT2 v 2.2.1 (11). Resulting files were sorted and indexed, and PCR and optical duplicate reads were marked using SamTools v1.12 (12). The numbers of reads mapping to each gene for each sample were counted using the R/Bioconductor package GenomicAlignments v 1.26.0 (13). Log_2_ fold change values were computed using DESeq2 version 1.30.1 (14). We used gene set enrichment analysis (GSEA), a pathway enrichment method that utilizes predefined gene sets from the reactome pathways (15), to analyze differentially expressed gene sets using the R/Bioconductor package fgsea v 1.16.0. The GSEA was performed to determine the key pathways that were enriched or inhibited by considering the expression levels of sets of biologically related genes (16). Genes identified by DESeq2 as expressing over a minimal threshold were ranked by Log_2_ fold change and analyzed by the GSEA algorithm (17). The altered pathways were filtered based on FDR ≤ 0.05 and were arranged in the order of their normalized enrichment scores.

## Results

The average RFI values of low- and high-RFI steers were−1.93 and 2.01, respectively. An average of 36 million reads per sample was generated (Supplementary Table 2). Results of GSEA revealed gene sets (pathways) associated with metabolism of proteins, cellular responses to external stimuli, stress, heat stress, and regulation of HSF-1-mediated heat shock response were differentially inhibited (FDR = 0.01) in high-RFI compared to low-RFI beef cattle (Table 1, Supplementary Figure 1). The gene set associated with metabolism of proteins consists of 248 genes, and 85 of which were leading edge genes (significantly enriched genes). Both cellular response to external stimuli and cellular response to stress shared the same nineteen leading edge genes including *HSPA1A, HSPH1, BAG2, DNAJA1, DNAJB1, H3C13, H2BC7, H4C2, ELOC, JUN*, and *HSPA4. Five* of the leading edge genes (*HSPA1A, HSPH1, BAG2, HSPA4*, and *DNAJB1*) associated with cellular response to external stimuli and stress were also leading edge genes in gene sets associated with response to heat stress and regulation of HSF-1-mediated heat shock response (Table 1).

**Table 1:** Altered pathways identified by Gene Set Enrichment Analysis in high-RFI compared to low-RFI beef steers.

**Pathway**	**FDR**	**NES**	**Gene set size (# of leading edge genes)**	**Leading edge genes**
Binding and uptake of ligands by scavenger receptors	0.01	1.82	4 (3)	*HBB, HBA1, HBA*
Scavenging of heme from plasma	0.01	1.82	4 (3)	*HBB, HBA1, HBA*
Erythrocytes take up carbon dioxide and release oxygen	0.01	1.68	3 (3)	*HBB, HBA1, HBA*
Erythrocytes take up oxygen and release carbon dioxide	0.01	1.68	3 (3)	*HBB, HBA1, HBA*
O_2_/CO_2_ exchange in erythrocytes	0.01	1.68	3 (3)	*HBB, HBA1, HBA*
Metabolism of proteins	0.01	−1.70	248 (85)	*LOC101907518, RPL39, LOC101902490, UBE2D1, FUCA2, B4GALT6, FBXL3, SOCS3, COMMD8, RPLP2, RPL34*
Cellular responses to external stimuli	0.01	−1.99	69 (19)	*HSPA1A, HSPH1, BAG2, DNAJA1, JUN, HSPA4, UBE2D1, DNAJB1, H3C13, H2BC7, H4C2, ELOC, ELOB, H2AC8, SIRT1, FLCN, ATP6V1G1, HSPA14, H2BU1*
Cellular responses to stress	0.01	−1.99	69 (19)	*HSPA1A, HSPH1, BAG2, DNAJA1, JUN, HSPA4, UBE2D1, DNAJB1, H3C13, H2BC7, H4C2, ELOC, ELOB, H2AC8, SIRT1, FLCN, ATP6V1G1, HSPA14, H2BU1*
Cellular response to heat stress	0.01	−2.05	16 (5)	*HSPA1A, HSPH1, BAG2, HSPA4, DNAJB1*
Regulation of HSF1-mediated heat shock response	0.01	−2.05	16 (5)	*HSPA1A, HSPH1, BAG2, HSPA4, DNAJB1*

*High-RFI, feed inefficient beef steers; low-RFI, feed-efficient beef steers; False discovery rate (FDR) ≤ 0.01; NES, normalized enrichment score (high-RFI vs. low-RFI). Leading edge genes are those that are enriched within the gene set. See Supplementary Table 3 for the full list of leading edge genes associated with metabolism of proteins. ^#^Means number*.

Gene sets associated with binding and uptake of ligands by scavenger receptors, scavenging of heme from plasma, erythrocytes take up/release carbon dioxide and release/take up oxygen share the same leading edge genes (*HBB, HBA1*, and *HBA*) and were all differentially enriched (FDR < 0.05) in high-RFI, relative to low-RFI beef cattle (Table 1, Supplementary Figure 2).

## Discussion

Understanding the biological mechanisms regulating feed efficiency using easily accessible and non-invasive sample such as blood is essential to the future of livestock production systems in terms of profitability and animal welfare concern.

In this study, protein metabolism is the most enriched metabolic pathway based on the number of leading edge genes (such as *LOC101907518, RPL39, LOC101902490, UBE2D1, FUCA2*) in the gene set. In addition to the function of amino acids as the building blocks of proteins, amino acids regulate key metabolism essential for growth, performance, reproduction, and immunity (18). Research studies have shown that protein (amino acids) metabolism is essential for optimizing efficiency of nutrient absorption and metabolism to enhance immunity against diseases and stress, growth performance, and milk production of animals (18). Several published articles have identified protein metabolism as one of the most important metabolic processes associated with RFI in animals (19, 20). In our previous study, we identified plasma amino acid metabolites as the most significant metabolic signatures associated with RFI in beef cattle (20). Elolimy et al. (21) reported differences in signaling mechanisms controlling protein turnover in ruminal epithelium of beef cattle divergent for low- or high-RFI. In a similar study, Kong et al. (3) reported increased expression of genes involved in protein and cell turnover in the ruminal epithelium of low-RFI beef cattle, compared with high-RFI beef cattle. 4 performed RNA-seq analysis of liver tissue in beef cattle divergent for low and high RFI and observed downregulation of genes involved in amino acid degradation and urea synthesis in low-RFI beef cattle. In fact, some studies have reported significant association of blood metabolites involved in urea cycle with RFI in beef cattle (22, 23). Our results and those of others that utilized tissues with relatively more invasive collection methods suggest that amino acid metabolism plays a considerable role in regulating RFI of beef cattle and its enrichment in low-RFI beef steers probably explains their similar growth performance with high-RFI beef steers despite lower DMI.

Amino acids play a functional role in regulating stress response, including oxidative stress, in animals (24). Stress response has significant implication on health and production efficiency of animals (25). In fact, difference in stress responsiveness has been suggested to contribute to variation in feed efficiency of beef cattle (19, 26). In this study, we observed downregulation of gene set including *HSPA1A, HSPH1, BAG2*, and *DNAJA1* associated with cellular responses to external stimuli, stress, heat stress, and regulation of HSF-1-mediated heat shock response in high-RFI beef steers, which suggests that these steers are more susceptible to stress. When an animal can no longer cope with a stressor, level of blood cortisol increases via activation of hypothalamic–pituitary–adrenal axis (HPA) axis which causes a fight or flight response that increases energy expenditure. Thus, stress response in animals is often determined by blood cortisol level and activity of the HPA axis (27). In a study that determined the response of beef heifers to an exogenous adrenocorticotropic hormone (ACTH) challenge, there was a positive association of plasma cortisol level with RFI status and low-RFI had a lower cortisol response than high-RFI heifers indicating that low-RFI heifers coped better with the stress challenge (28). Richardson et al. (19) and Gomes et al. (29) reported lower blood levels of cortisol in low-RFI beef cattle when compared to high-RFI beef cattle. A similar result was observed in crossbred rams following ACTH challenge (26). Taken together, downregulation of genes associated with cellular responses to external stimuli, stress, heat stress, and regulation of HSF-1-mediated heat shock response in high-RFI beef steers suggests that low-RFI steers have better adaptive mechanisms to cope with environmental stressors, thereby, reducing energy expenditure and increasing energy availability for improved growth performance and better feed efficiency.

In this study, we observed enrichment of gene sets (*HBB, HBA1*, and *HBA*) associated with erythrocytes take up/release carbon dioxide, release/take up oxygen, scavenging of heme from plasma, and binding and uptake of ligands by scavenger receptors in high-RFI, relative to low-RFI beef cattle. Erythrocytes contain hemoglobins which carry oxygen to the body and are continuously exposed to high oxygen content which pre-disposes them to oxidative stress damage (30, 31). Heme scavenger proteins, such as hemopexin and alpha-1-microglobulin, scavenge extracellular heme, a physiological ligand (32), synthesized from hemoglobin degradation via the activity of heme-oxygenase, an enzyme that is inducible by stressors such as oxygen free radicals (33, 34). Previous investigations have shown that cellular expression of alpha-1-microglobulin is enriched during increased oxidative stress and heme exposure (30, 34). In ruminants, oxidative stress has been implicated in many pathophysiological conditions that are relevant for growth performance, reproduction, and health (35). In fact, several studies have shown that oxidative damage of cell organelles and biomolecules is a source of energy drain and negatively affects several cellular processes including lipid and protein metabolism (36, 37). The major source of intracellular reactive oxygen species production is the mitochondria (38) and previous studies have reported higher mitochondrial ROS production in less feed-efficient compared to high feed efficient animals (36, 39, 40). In addition, Casal et al. (41) reported increased hepatic abundance of protein carbonyls and thiobarbituric acid reactive species, which are products of protein and lipid oxidative damage, and reduced protein expression of antioxidant enzymes, including mitochondrial manganese superoxide dismutase, in high-RFI when compared with low-RFI beef steers. Similarly, Tizioto et al. (42) observed upregulation of oxidative stress-induced transcription factors in muscle of high-RFI beef steers. Therefore, it is reasonable to speculate that enrichment of genes associated with erythrocytes take up/release carbon dioxide, scavenging of heme from plasma, and binding and uptake of ligands by scavenger receptors in high-RFI compared to low-RFI beef steers suggests that they may be more prone to oxidative stress, thereby resulting in reduced efficiency of energy use for metabolic processes such as growth.

It is important to note that though whole blood transcriptome data might encompass gene activities of several body tissues and organs including liver, kidney, muscles, and rumen, the contribution of each tissue to the whole blood transcriptome is not known and should be determined in future studies. In addition, biological validation of the RNA-Seq data on selected genes by RT-qPCR is also needed to confirm the results of this study.

## Conclusion

Results of GSEA of whole blood transcriptome data in beef steers divergently selected for low or high RFI revealed differential expression of genes related to protein metabolism, erythrocytes take up/release carbon dioxide and release/take up oxygen, and stress responsiveness. These results are similar to those of several studies that utilized other tissues including liver, muscle, and ruminal epithelium. Thus, this study demonstrates the suitability of whole blood transcriptome data for understanding the biological mechanisms regulating RFI in animals. Due to the small number of animals used in this study and the effect of different diets and breeds on RFI ranking, further validation using a larger cohort of beef cattle fed different diets is needed to confirm these findings.

## Data Availability Statement

All raw and processed sequencing data were submitted to the Gene Expression Omnibus (GEO) at the National Center for Biotechnology Information (NCBI) and can be accessed via accession number GSE198068.

## Ethics Statement

The animal study was reviewed and approved by the Institutional Animal Care and Use Committees of West Virginia University (protocol number 1608003693).

## Author Contributions

IO and APC designed the experiment. GT and MI conducted the experiment. JD analyzed the RNA-Seq data. IO, GT, and APC drafted the manuscript. All authors reviewed and approved the final manuscript.

## Funding

This work was funded by West Virginia University Experimental Station (scientific article number 3430) in support of U.S. Department of Agriculture hatch multi-state regional project W-3010.

## Conflict of Interest

The authors declare that the research was conducted in the absence of any commercial or financial relationships that could be construed as a potential conflict of interest.

## Publisher's Note

All claims expressed in this article are solely those of the authors and do not necessarily represent those of their affiliated organizations, or those of the publisher, the editors and the reviewers. Any product that may be evaluated in this article, or claim that may be made by its manufacturer, is not guaranteed or endorsed by the publisher.
