# Supplementary material for: Identification of Key Pathways Associated With Residual Feed Intake of Beef Cattle Based on Whole Blood Transcriptome Data Analyzed Using Gene Set Enrichment Analysis
Source: Front Vet Sci. 2022 Apr 18;9:848027. doi: 10.3389/fvets.2022.848027 (PMC9062580; doi:10.3389/fvets.2022.848027)
Supplement: Supplementary file 1 [file Image_1.pdf]

A.

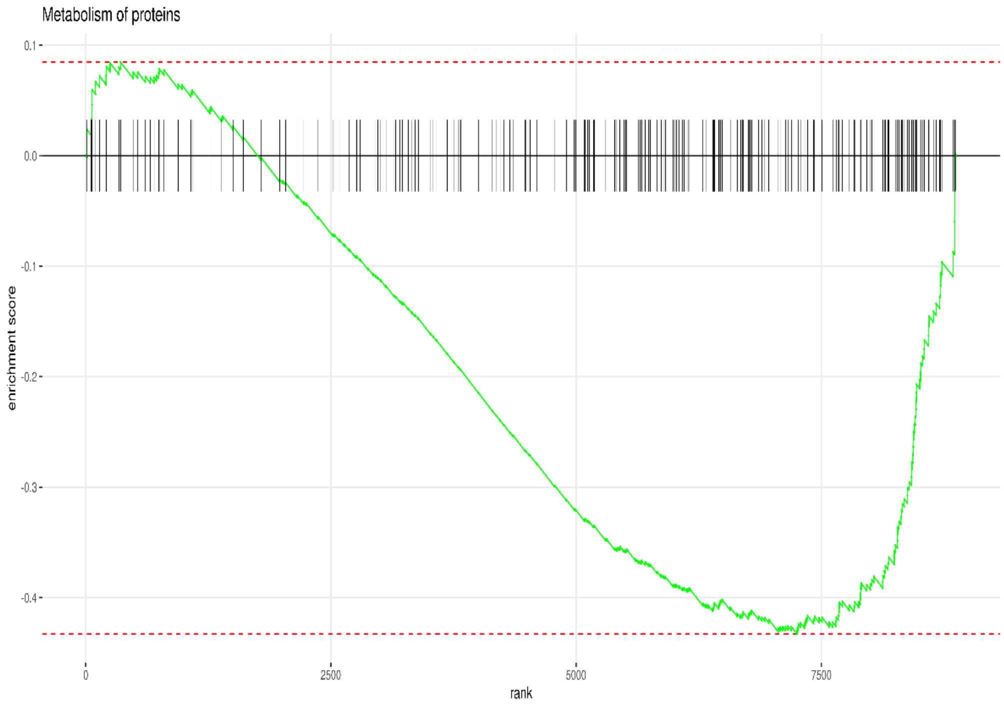
Metabolism of proteins
0.1
0.0
-0.1
-0.2
-0.3
-0.4
enrichment score
0
2500
5000
7500
rank

B.

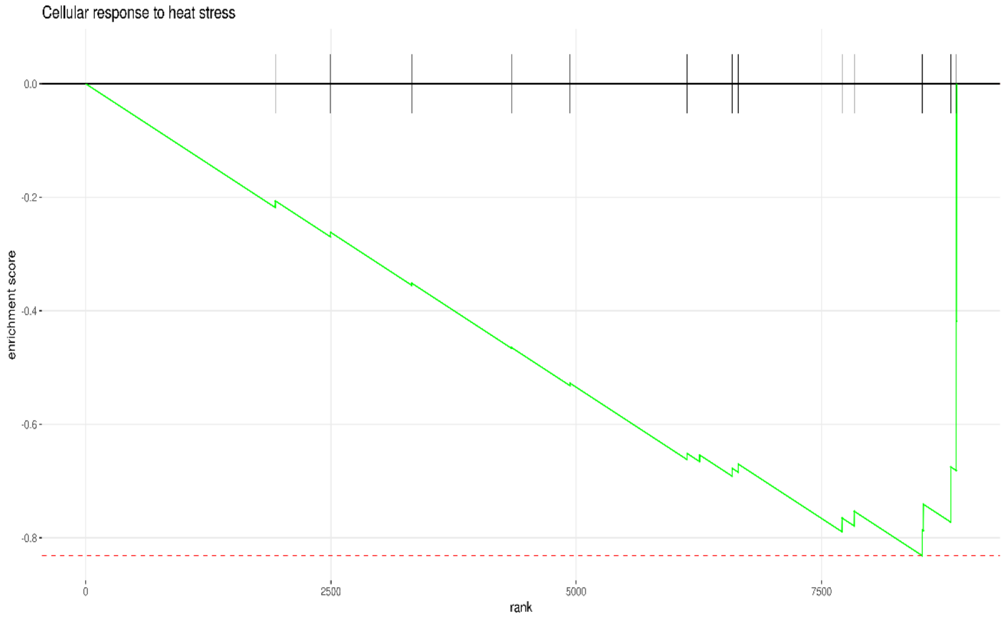
Cellular response to heat stress
0.0
-0.2
-0.4
-0.6
-0.8
enrichment score
0
2500
5000
7500
rank

C.

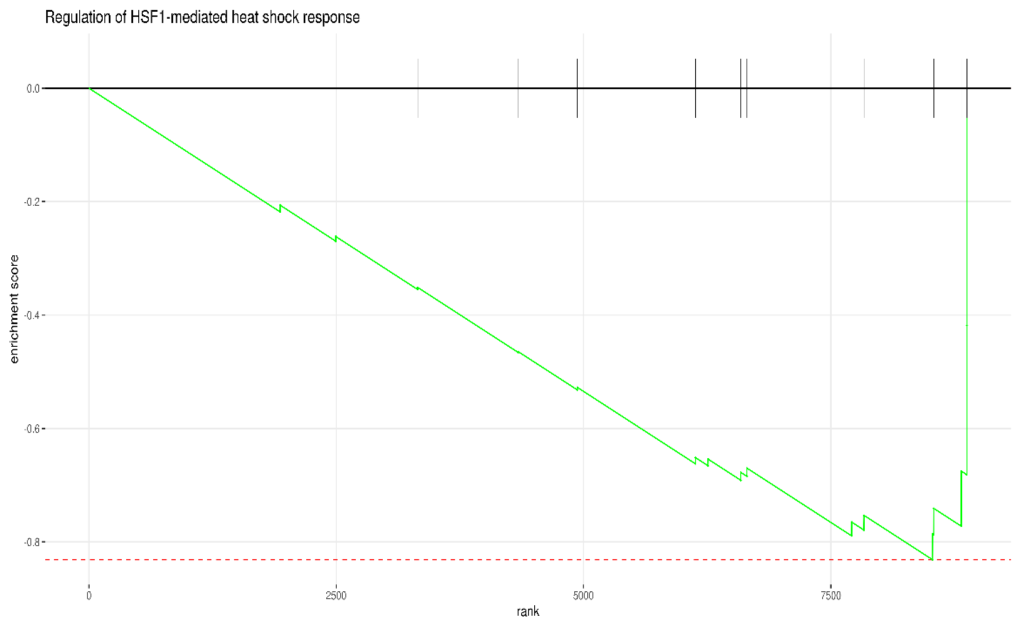

Regulation of HSF1-mediated heat shock response
0.0
-0.2
-0.4
-0.6
-0.8
enrichment score
0
2500
5000
7500
rank

D.

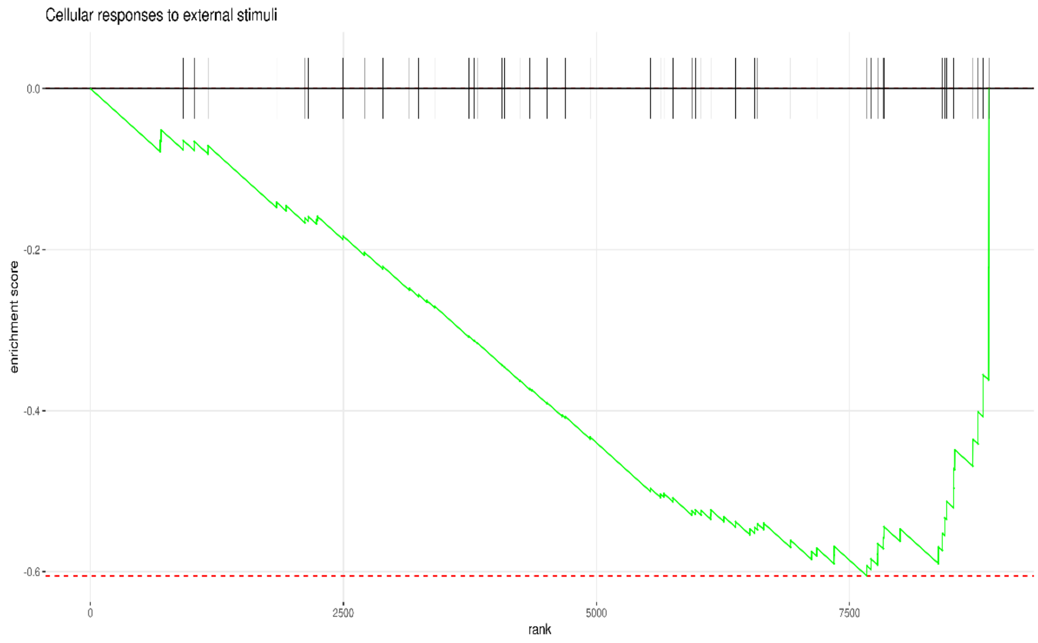

Cellular responses to external stimuli
0.0
-0.2
-0.4
-0.6
enrichment score
0
2500
5000
7500
rank

E.

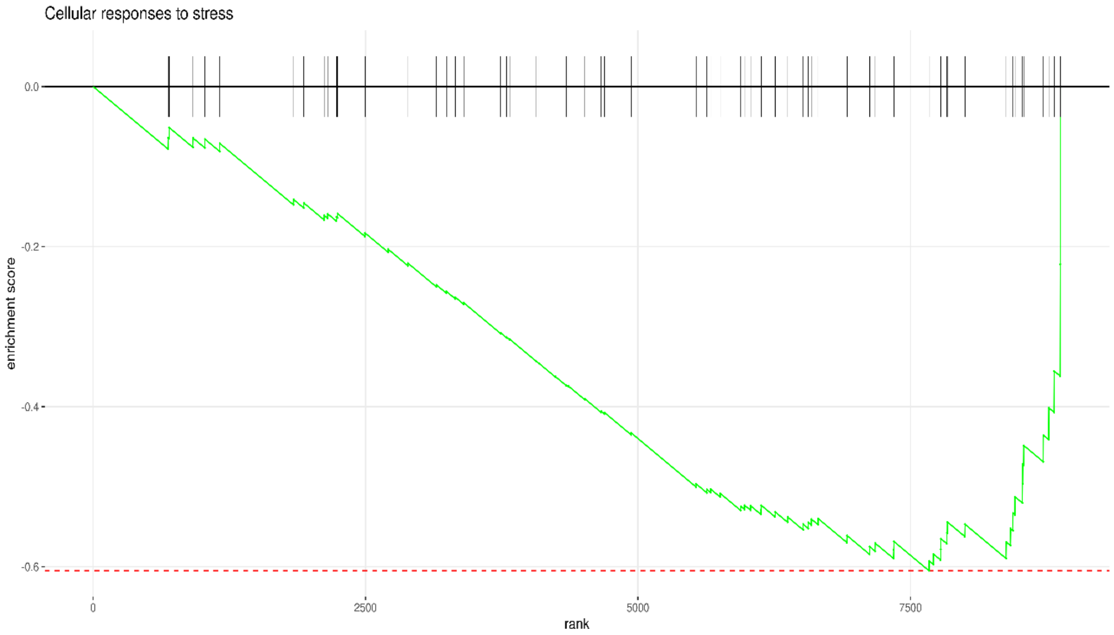

Figure S1. Gene set enrichment analysis (GSEA)-enrichment plots of representative pathways inhibited in high-RFI compared to low-RFI beef steers; reactome metabolism of proteins (A), cellular response to heat stress (B), regulation of HSF1-mediated heat shock response (C), cellular response to external stimuli (D), and cellular response to stress (E).
