# Supplementary material for: Identification of Key Pathways Associated With Residual Feed Intake of Beef Cattle Based on Whole Blood Transcriptome Data Analyzed Using Gene Set Enrichment Analysis
Source: Front Vet Sci. 2022 Apr 18;9:848027. doi: 10.3389/fvets.2022.848027 (PMC9062580; doi:10.3389/fvets.2022.848027)
Supplement: Supplementary file 2 [file Image_2.pdf]

A.

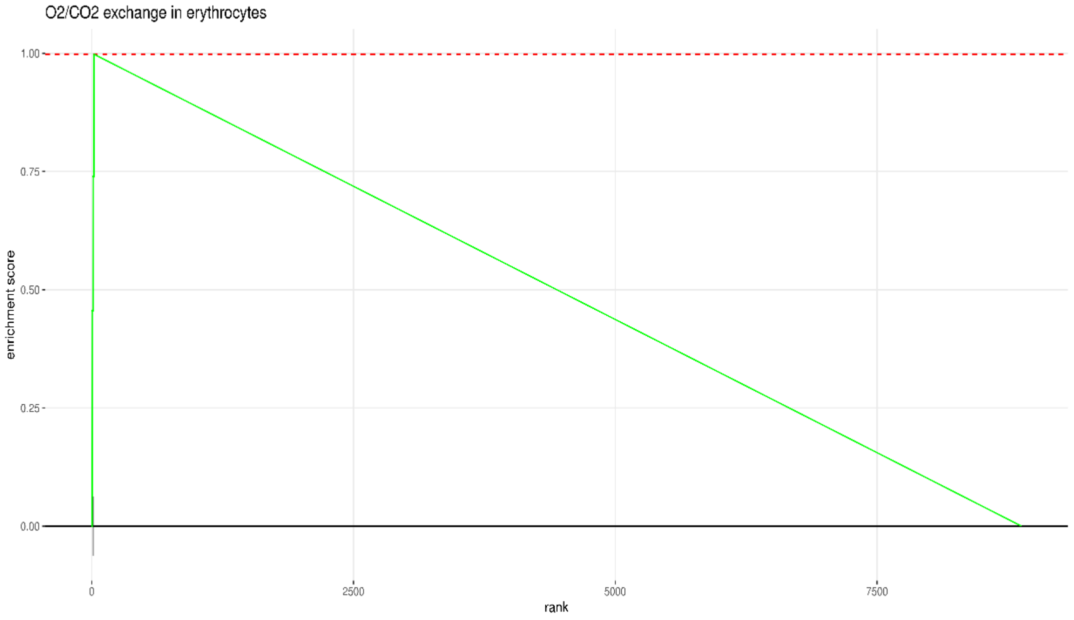
O2/CO2 exchange in erythrocytes
1.00
0.75
0.50
0.25
0.00
enrichment score
0
2500
5000
7500
rank

B.

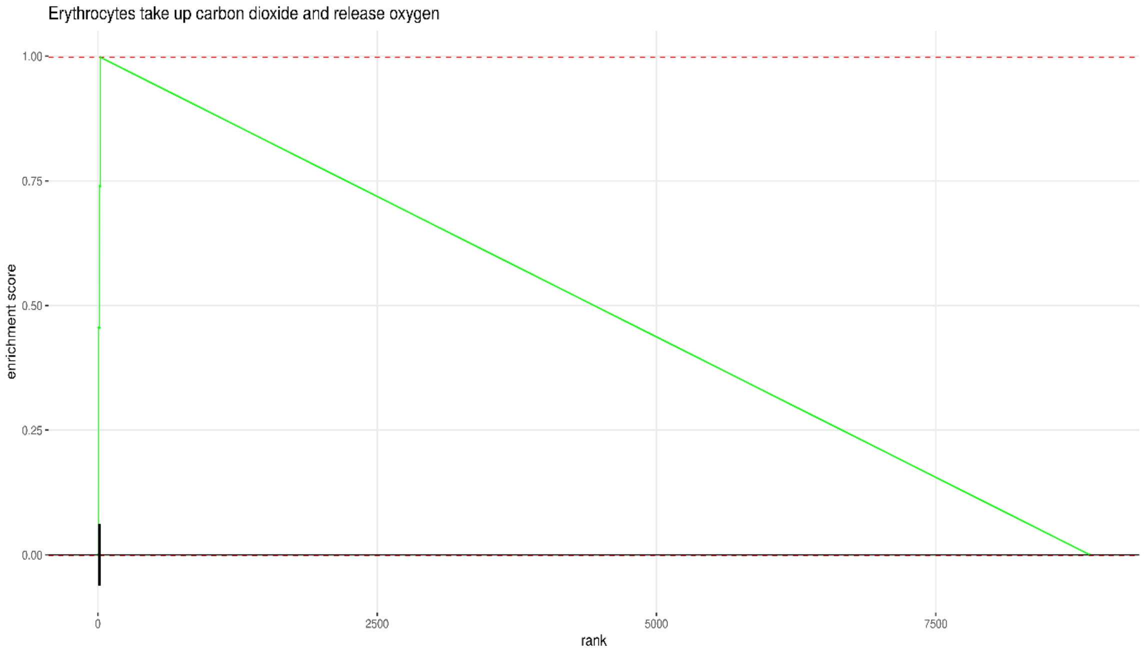
Erythrocytes take up carbon dioxide and release oxygen
1.00
0.75
0.50
0.25
0.00
enrichment score
0
2500
5000
7500
rank

C.

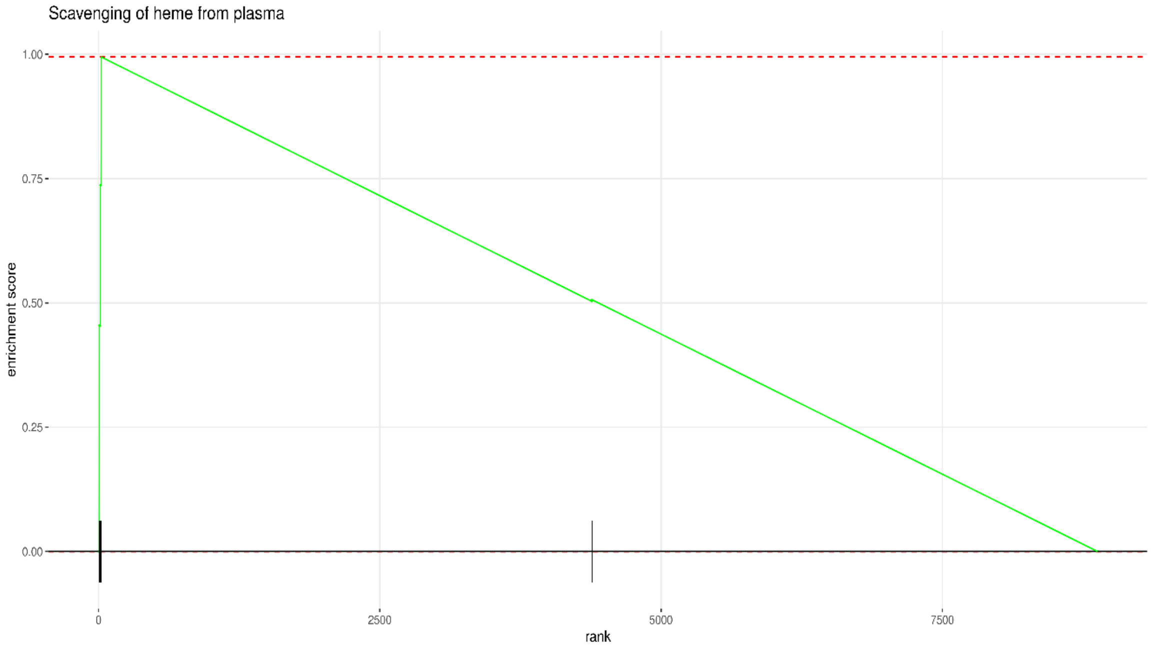

D.

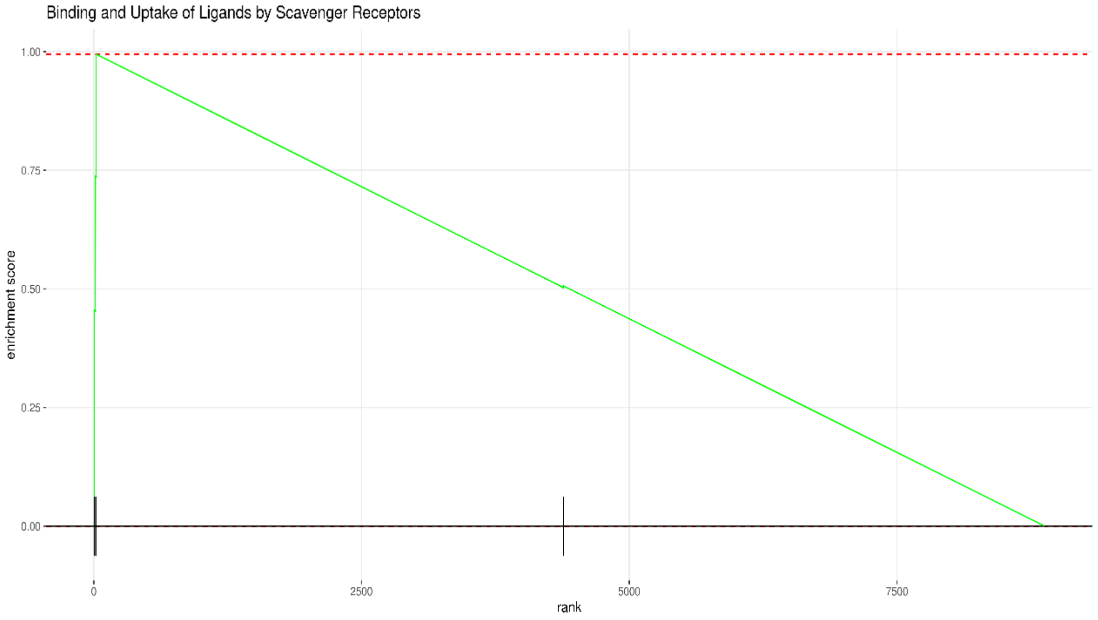

Figure 2. Gene set enrichment analysis (GSEA)-enrichment plots of representative pathways enriched in high-RFI compared to low-RFI beef steers; O_2/CO_2 exchange in erythrocytes (A), erythrocytes take up carbon dioxide and release oxygen (B), scavenging of heme from plasma (C), and binding and uptake of ligands by scavenger receptors cellular response to external stimuli (D).
