# Supplementary material for: Identification of Key Pathways Associated With Residual Feed Intake of Beef Cattle Based on Whole Blood Transcriptome Data Analyzed Using Gene Set Enrichment Analysis
Source: Front Vet Sci. 2022 Apr 18;9:848027. doi: 10.3389/fvets.2022.848027 (PMC9062580; doi:10.3389/fvets.2022.848027)
Supplement: Supplementary file 3 [file Table_1.docx]

Table S1. Ingredient and nutrient composition of the basal diet^1^

| **Item** | **Value**^2^ |
| --- | --- |
| Ingredient composition |  |
| Triticale silage | 49.5 |
| Ryegrass silage | 47.5 |
| Concentrate supplement^3^ | 3.0 |
| Nutrient composition^4^ | |
| DM, % | 44.5 |
| CP | 13.7 |
| aNDF | 59.7 |
| ADF | 31.5 |
| EE | 3.14 |
| Ca | 0.66 |
| P | 0.37 |
| NE_m_, Mcal/kg | 1.37 |
| NE_g_, Mcal/kg | 0.91 |

^1^Composition of basal diet calculated from analysis and concentration of individual ingredients.

^2^Values are presented on a % DM basis unless indicated otherwise.

^3^Traditions 50% beef supplement (Southern States Cooperative, Richmond, VA) contained processed grain by-products, plant protein products, ground limestone, urea, salt, cane molasses, potassium sulfate, magnesium sulfate, sodium selenite, vitamin A supplement, calcium carbonate, vegetable oil, manganous oxide, vitamin D3 supplement, vitamin E supplement, zinc oxide, lecithin, phosphoric acid, basic copper chloride, magnesium chloride, propylene glycol, natural and artificial flavors, ferrous sulfate, calcium iodate, and cobalt carbonate; Guaranteed analysis: 50% CP; 5% Ca; 0.55% P; 2% Na; 3.9% salt; 1% K, and 66,000 IU/kg vitamin A.

^4^DM = dry matter; CP = crude protein; aNDF = neutral detergent fiber (amylase treated); ADF = acid detergent fiber; EE = ether extract; NE_m_ = net energy of maintenance; NE_g_ = net energy of gain.
